# CRNPRED: highly accurate prediction of one-dimensional protein structures by large-scale critical random networks

**DOI:** 10.1186/1471-2105-7-401

**Published:** 2006-09-05

**Authors:** Akira R Kinjo, Ken Nishikawa

**Affiliations:** 1 Center for Information Biology and DNA Data Bank of Japan, National Institute of Genetics, Mishima, 411-8540, Japan; 2 Department of Genetics, The Graduate University for Advanced Studies (SOKENDAI), Mishima 411-8540, Japan; 3 Research Center for Structural and Functional Proteomics, Institute for Protein Research, Osaka University, 3-2 Suita, 565-0871, Japan

## Abstract

**Background:**

One-dimensional protein structures such as secondary structures or contact numbers are useful for three-dimensional structure prediction and helpful for intuitive understanding of the sequence-structure relationship. Accurate prediction methods will serve as a basis for these and other purposes.

**Results:**

We implemented a program CRNPRED which predicts secondary structures, contact numbers and residue-wise contact orders. This program is based on a novel machine learning scheme called critical random networks. Unlike most conventional one-dimensional structure prediction methods which are based on local windows of an amino acid sequence, CRNPRED takes into account the whole sequence. CRNPRED achieves, on average per chain, *Q*_3 _= 81% for secondary structure prediction, and correlation coefficients of 0.75 and 0.61 for contact number and residue-wise contact order predictions, respectively.

**Conclusion:**

CRNPRED will be a useful tool for computational as well as experimental biologists who need accurate one-dimensional protein structure predictions.

## Background

One-dimensional (1D) structures of a protein are residue-wise quantities or symbols onto which some features of the native three-dimensional (3D) structure are projected. 1D structures are of interest for several reasons. For example, predicted secondary structures, a kind of 1D structures, are often used to limit the conformational space to be searched in 3D structure prediction. Furthermore, it has recently been shown that certain sets of the native (as opposed to predicted) 1D structures of a protein contain sufficient information to recover the native 3D structure [1,2]. These 1D structures are either the principal eigenvector of the contact map [1] or a set of secondary structures (SS), contact numbers (CN) and residue-wise contact orders (RWCO) [2]. Therefore, it is possible, at least in principle, to predict the native 3D structure by first predicting the 1D structures, and then by constructing the 3D structure from these 1D structures. 1D structures are not only useful for 3D structure predictions, but also helpful for intuitive understanding of the correspondence between the protein structure and its amino acid sequence due to the residue-wise characteristics of 1D structures. Therefore, accurate prediction of 1D protein structures is of fundamental biological interest.

Secondary structure prediction has a long history [3]. Almost all the modern predictors are based on position-specific scoring matrices (PSSM) and some kind of machine learning techniques such as neural networks or support vector machines. Currently the best predictors achieve *Q*_3 _of 77–79% [4,5]. The study of contact number prediction also started long time ago [6,7], but further improvements were made only recently [8-10]. These recent methods are based on the ideas developed in SS predictions (i.e., PSSM and machine learning), and achieve a correlation coefficient of 0.68–0.73.

Recently, we have developed a new method for accurately predicting SS, CN, and RWCO based on a novel machine learning scheme, critical random networks (CRN) [10]. In this paper, we briefly describe the formulation of the method, and recent improvements leading to even better predictions. The computer program for SS, CN, and RWCO prediction named CRNPRED has been developed for the convenience of the general user, and a web interface and source code are made available online.

## Implementation

### Definition of 1D structures

#### Secondary structures (SS)

Secondary structures were defined by the DSSP program [11]. For three-state SS prediction, the simple encoding scheme (the so-called CK mapping) was employed [12]. That is, *α *helices (*H*), *β *strands (*E*), and other structures ("coils") defined by DSSP were encoded as *H*, *E*, and *C*, respectively. Note that we do not use the CASP-style conversion scheme (the so-called EHL mapping) in which DSSP's *H*, *G *(3_10 _helix) and *I *(*π *helix) are encoded as *H*, and DSSP's *E *and *B *(*β *bridge) as *E*. We believe the CK mapping is more natural and useful for 3D structure predictions (e.g., geometrical restraints should be different between an *α *helix and a 3_10 _helix). For SS prediction, we introduce feature variables (yiH,yiE,yiC
 MathType@MTEF@5@5@+=feaafiart1ev1aaatCvAUfKttLearuWrP9MDH5MBPbIqV92AaeXatLxBI9gBaebbnrfifHhDYfgasaacH8akY=wiFfYdH8Gipec8Eeeu0xXdbba9frFj0=OqFfea0dXdd9vqai=hGuQ8kuc9pgc9s8qqaq=dirpe0xb9q8qiLsFr0=vr0=vr0dc8meaabaqaciaacaGaaeqabaqabeGadaaakeaacqWG5bqEdaqhaaWcbaGaemyAaKgabaGaemisaGeaaOGaeiilaWIaemyEaK3aa0baaSqaaiabdMgaPbqaaiabdweafbaakiabcYcaSiabdMha5naaDaaaleaacqWGPbqAaeaacqWGdbWqaaaaaa@3AC4@) to represent each type of secondary structures at the *i*-th residue position, so that *H *is represented as (1, -1, -1), *E *as (-1, 1, -1), and *C *as (-1, -1, 1).

#### Contact numbers (CN)

Let *C*_*i*,*j *_represent the contact map of a protein. Usually, the contact map is defined so that *C*_*i*,*j *_= 1 if the *i*-th and *j*-th residues are in contact by some definition, or *C*_*i*,*j *_= 0, otherwise. As in our previous study, we slightly modify the definition using a sigmoid function. That is,

*C*_*i*,*j *_= 1/{1 + exp [*w*(*r*_*i*,*j *_- *d*)]}     (1)

where *r*_*i*,*j *_is the distance between *C*_*β *_(*C*_*α *_for glycines) atoms of the *i*-th and *j*-th residues, *d *= 12Å is a cutoff distance, and *w *is a sharpness parameter of the sigmoid function which is set to 3 [8,2]. The rather generous cutoff length of 12Å was shown to optimize the prediction accuracy [8]. The use of the sigmoid function enables us to use the contact numbers in molecular dynamics simulations [2]. Using the above definition of the contact map, the contact number of the *i*-th residue of a protein is defined as

ni=∑j:|i−j|>2Ci,j.     (2)
 MathType@MTEF@5@5@+=feaafiart1ev1aaatCvAUfKttLearuWrP9MDH5MBPbIqV92AaeXatLxBI9gBaebbnrfifHhDYfgasaacH8akY=wiFfYdH8Gipec8Eeeu0xXdbba9frFj0=OqFfea0dXdd9vqai=hGuQ8kuc9pgc9s8qqaq=dirpe0xb9q8qiLsFr0=vr0=vr0dc8meaabaqaciaacaGaaeqabaqabeGadaaakeaacqWGUbGBdaWgaaWcbaGaemyAaKgabeaakiabg2da9maaqafabaGaem4qam0aaSbaaSqaaiabdMgaPjabcYcaSiabdQgaQbqabaaabaGaemOAaOMaeiOoaOZaaqWaceaacqWGPbqAcqGHsislcqWGQbGAaiaawEa7caGLiWoacqGH+aGpcqaIYaGmaeqaniabggHiLdGccqGGUaGlcaWLjaGaaCzcamaabmGabaGaeGOmaidacaGLOaGaayzkaaaaaa@475E@

The feature variable *y*_*i *_for CN is defined as *y*_*i *_= *n*_*i*_/log *L *where *L *is the sequence length of a target protein. The normalization factor log *L *is introduced because we have observed that the contact number averaged over a protein chain is roughly proportional to log *L*, and thus division by this value removes the size-dependence of predicted contact numbers.

#### Residue-wise contact orders (RWCO)

RWCO was first introduced in [2]. This quantity measures the extent to which a residue makes long-range contacts in a native protein structure. Using the same notation as contact numbers, the RWCO of the *i*-th residue in a protein structure is defined by

oi=∑j:|i−j|>2|i−j|Ci,j.     (3)
 MathType@MTEF@5@5@+=feaafiart1ev1aaatCvAUfKttLearuWrP9MDH5MBPbIqV92AaeXatLxBI9gBaebbnrfifHhDYfgasaacH8akY=wiFfYdH8Gipec8Eeeu0xXdbba9frFj0=OqFfea0dXdd9vqai=hGuQ8kuc9pgc9s8qqaq=dirpe0xb9q8qiLsFr0=vr0=vr0dc8meaabaqaciaacaGaaeqabaqabeGadaaakeaacqWGVbWBdaWgaaWcbaGaemyAaKgabeaakiabg2da9maaqafabaWaaqWaceaacqWGPbqAcqGHsislcqWGQbGAaiaawEa7caGLiWoaaSqaaiabdQgaQjabcQda6maaemGabaGaemyAaKMaeyOeI0IaemOAaOgacaGLhWUaayjcSdGaeyOpa4JaeGOmaidabeqdcqGHris5aOGaem4qam0aaSbaaSqaaiabdMgaPjabcYcaSiabdQgaQbqabaGccqGGUaGlcaWLjaGaaCzcamaabmGabaGaeG4mamdacaGLOaGaayzkaaaaaa@4E40@

The feature variable *y*_*i *_for RWCO is defined as *y*_*i *_= *o*_*i*_/*L *where *L *is the sequence length. Due to the similar reason as CN, the normalization factor *L *was introduced to remove the size-dependence of the predicted RWCOs (the RWCO averaged over a protein chain is roughly proportional to the chain length).

### Critical random networks

Here we briefly describe the critical random network (CRN) method introduced in [10] which should be referred to for the details. Unlike most conventional methods for 1D structure prediction [except for some including the bidirectional recurrent neural networks [13, 5, 14]], the CRN method takes the whole amino acid sequence into account. In the CRN method, an *N*-dimensional state vector **x**_*i *_is assigned to the *i*-th residue of the target sequence (we use *N *= 5000 throughout this paper). Neighboring state vectors along the sequence are connected via a random *N *× *N *orthogonal matrix *W*. This matrix is also block-diagonal with the size of blocks ranging uniformly randomly between 2 and 50. The input to the CRN is the position-specific scoring matrix (PSSM), *U *= (**u**_1_, ..., **u**_*L*_) of the target sequence obtained by PSI-BLAST [15] (*L *is the sequence length of the target protein). We impose that the state vectors satisfy the following equation of state:

**x**_*i *_= tanh [*βW *(**x**_*i*-1 _+ **x**_*i*+1_) + *αV***u**_*i*_]     (4)

for *i *= 1, ..., *L *where *V *is an *N *× 21 random matrix (the 21st component of **u**_*i *_is always set to unity), and *β *and *α *are scalar parameters. The fixed boundary condition is imposed (**x**_0 _= **x**_*L*+1 _= **0**). By setting *β *= 0.5, the system of state vectors is made to be near a critical point in a certain sense, and thus the range of site-site correlation is expected to be long when *α *is sufficiently small but finite [10]. The value of *α *was chosen so that the resulting solution **x**_*i *_oscillates continuously with respect to the residue number *i*; that is, each component of **x**_*i *_having values from -1 to 1, rather than being a discrete sequence of -1 or 1. It can be shown that there exists a unique solution of Eq. 4 for a given PSSM *U *(provided the above boundary condition and *β *= 0.5). The solution {**x**_*i*_} of Eq. 4 (i.e., the state vectors) can be interpreted as some kind of patterns that reflect the complicated interactions among neighboring residues along the amino acid sequence. In this way, each state vector implicitly incorporates long-range correlations, and its components serve as additional independent variables to the linear predictor described in the following. The 1D structure of the *i*-th residue is predicted as a linear projection of a local window of the PSSM and the state vector obtained by solving Eq. 4:

yi=∑m=−MM∑a=121Dm,aua,i+m+∑k=1NEkxk,i     (5)
 MathType@MTEF@5@5@+=feaafiart1ev1aaatCvAUfKttLearuWrP9MDH5MBPbIqV92AaeXatLxBI9gBaebbnrfifHhDYfgasaacH8akY=wiFfYdH8Gipec8Eeeu0xXdbba9frFj0=OqFfea0dXdd9vqai=hGuQ8kuc9pgc9s8qqaq=dirpe0xb9q8qiLsFr0=vr0=vr0dc8meaabaqaciaacaGaaeqabaqabeGadaaakeaacqWG5bqEdaWgaaWcbaGaemyAaKgabeaakiabg2da9maaqahabaWaaabCaeaacqWGebardaWgaaWcbaGaemyBa0MaeiilaWIaemyyaegabeaakiabdwha1naaBaaaleaacqWGHbqycqGGSaalcqWGPbqAcqGHRaWkcqWGTbqBaeqaaaqaaiabdggaHjabg2da9iabigdaXaqaaiabikdaYiabigdaXaqdcqGHris5aaWcbaGaemyBa0Maeyypa0JaeyOeI0Iaemyta0eabaGaemyta0eaniabggHiLdGccqGHRaWkdaaeWbqaaiabdweafnaaBaaaleaacqWGRbWAaeqaaOGaemiEaG3aaSbaaSqaaiabdUgaRjabcYcaSiabdMgaPbqabaaabaGaem4AaSMaeyypa0JaeGymaedabaGaemOta4eaniabggHiLdGccaWLjaGaaCzcamaabmGabaGaeGynaudacaGLOaGaayzkaaaaaa@5F8A@

where *y*_*i *_is the predicted quantity, and *D*_*m*,*a *_and *E*_*k *_are the regression parameters. In the first summation, each PSSM column is extended to include the "terminal" residue. Since Eq. 5 is a simple linear equation once the equation of state (Eq. 4) has been solved, learning the parameters *D*_*m*,*a *_and *E*_*k *_reduces to an ordinary linear regression problem. For SS prediction, the triple (yiH,yiE,yiC
 MathType@MTEF@5@5@+=feaafiart1ev1aaatCvAUfKttLearuWrP9MDH5MBPbIqV92AaeXatLxBI9gBaebbnrfifHhDYfgasaacH8akY=wiFfYdH8Gipec8Eeeu0xXdbba9frFj0=OqFfea0dXdd9vqai=hGuQ8kuc9pgc9s8qqaq=dirpe0xb9q8qiLsFr0=vr0=vr0dc8meaabaqaciaacaGaaeqabaqabeGadaaakeaacqWG5bqEdaqhaaWcbaGaemyAaKgabaGaemisaGeaaOGaeiilaWIaemyEaK3aa0baaSqaaiabdMgaPbqaaiabdweafbaakiabcYcaSiabdMha5naaDaaaleaacqWGPbqAaeaacqWGdbWqaaaaaa@3AC4@) is calculated simultaneously, and the SS class is predicted as arg max_*s*∈{*H*,*E*,*C*}_yis
 MathType@MTEF@5@5@+=feaafiart1ev1aaatCvAUfKttLearuWrP9MDH5MBPbIqV92AaeXatLxBI9gBaebbnrfifHhDYfgasaacH8akY=wiFfYdH8Gipec8Eeeu0xXdbba9frFj0=OqFfea0dXdd9vqai=hGuQ8kuc9pgc9s8qqaq=dirpe0xb9q8qiLsFr0=vr0=vr0dc8meaabaqaciaacaGaaeqabaqabeGadaaakeaacqWG5bqEdaqhaaWcbaGaemyAaKgabaGaem4Camhaaaaa@311E@. For the CN and RWCO prediction, real values are predicted. 2-state prediction is also made for CN using the average CN for each residue type as the threshold for "exposed" or "buried" as in [16]. We have noted earlier [8] that the apparent accuracy of 2-state CN prediction depend on the threshold. Although we proposed that using the median instead of average CN should be more appropriate for the threshold, here we use the average in order to compare our results with others. The half window size *M *is set to 9 for SS and CN predictions, and to 26 for RWCO. Note that the solution of the equation of state (Eq. 4) is determined solely by the PSSM. Therefore, obtaining the solution to Eq. (4) can be regarded as a kind of unsupervised learning, and the method for solving the equation of state is irrelevant for learning the parameters.

### Ensemble prediction

Since the CRN-based prediction is parametrized by the random matrices *W *and *V*, slightly different predictions are obtained for different pairs of *W *and *V*. We can improve the prediction by taking the average over an ensemble of such different predictions. 20 CRN-based predictors were constructed using 20 sets of different random matrices *W *and *V*. CN and RWCO are predicted as uniform averages of these 20 predictions.

For SS prediction, we employ further training. Let sit,n
 MathType@MTEF@5@5@+=feaafiart1ev1aaatCvAUfKttLearuWrP9MDH5MBPbIqV92AaeXatLxBI9gBaebbnrfifHhDYfgasaacH8akY=wiFfYdH8Gipec8Eeeu0xXdbba9frFj0=OqFfea0dXdd9vqai=hGuQ8kuc9pgc9s8qqaq=dirpe0xb9q8qiLsFr0=vr0=vr0dc8meaabaqaciaacaGaaeqabaqabeGadaaakeaacqWGZbWCdaqhaaWcbaGaemyAaKgabaGaemiDaqNaeiilaWIaemOBa4gaaaaa@3359@ be the prediction results of the *n*-th predictor for 1D structure *t *(*H*, *E*, *C*, CN, and RWCO) of the *i*-th residue. The second stage SS prediction is made by the following linear scheme:

yiss=∑n=120∑t∑m=−33wn,t,msi+mt,n     (6)
 MathType@MTEF@5@5@+=feaafiart1ev1aaatCvAUfKttLearuWrP9MDH5MBPbIqV92AaeXatLxBI9gBaebbnrfifHhDYfgasaacH8akY=wiFfYdH8Gipec8Eeeu0xXdbba9frFj0=OqFfea0dXdd9vqai=hGuQ8kuc9pgc9s8qqaq=dirpe0xb9q8qiLsFr0=vr0=vr0dc8meaabaqaciaacaGaaeqabaqabeGadaaakeaacqWG5bqEdaqhaaWcbaGaemyAaKgabaGaem4CamNaem4CamhaaOGaeyypa0ZaaabCaeaadaaeqbqaamaaqahabaGaem4DaC3aaSbaaSqaaiabd6gaUjabcYcaSiabdsha0jabcYcaSiabd2gaTbqabaGccqWGZbWCdaqhaaWcbaGaemyAaKMaey4kaSIaemyBa0gabaGaemiDaqNaeiilaWIaemOBa4gaaaqaaiabd2gaTjabg2da9iabgkHiTiabiodaZaqaaiabiodaZaqdcqGHris5aaWcbaGaemiDaqhabeqdcqGHris5aaWcbaGaemOBa4Maeyypa0JaeGymaedabaGaeGOmaiJaeGimaadaniabggHiLdGccaWLjaGaaCzcamaabmGabaGaeGOnaydacaGLOaGaayzkaaaaaa@5A8E@

where *ss *= *H*, *E*, *C*, and *w*_*n*,*t*,*m *_is the weight obtained from a training set. Finally, the feature variable for each SS class of the *i*-th residue is obtained by (yi−1ss+2yiss+yi+1ss
 MathType@MTEF@5@5@+=feaafiart1ev1aaatCvAUfKttLearuWrP9MDH5MBPbIqV92AaeXatLxBI9gBaebbnrfifHhDYfgasaacH8akY=wiFfYdH8Gipec8Eeeu0xXdbba9frFj0=OqFfea0dXdd9vqai=hGuQ8kuc9pgc9s8qqaq=dirpe0xb9q8qiLsFr0=vr0=vr0dc8meaabaqaciaacaGaaeqabaqabeGadaaakeaacqWG5bqEdaqhaaWcbaGaemyAaKMaeyOeI0IaeGymaedabaGaem4CamNaem4CamhaaOGaey4kaSIaeGOmaiJaemyEaK3aa0baaSqaaiabdMgaPbqaaiabdohaZjabdohaZbaakiabgUcaRiabdMha5naaDaaaleaacqWGPbqAcqGHRaWkcqaIXaqmaeaacqWGZbWCcqWGZbWCaaaaaa@44C8@)/4. This last procedure was found particularly effective for improving the segment overlap (SOV) measure.

### Additional input

Another improvement is the addition of the amino acid composition of the target sequence to the predictor [9]: The term ∑a=120Fafa
 MathType@MTEF@5@5@+=feaafiart1ev1aaatCvAUfKttLearuWrP9MDH5MBPbIqV92AaeXatLxBI9gBaebbnrfifHhDYfgasaacH8akY=wiFfYdH8Gipec8Eeeu0xXdbba9frFj0=OqFfea0dXdd9vqai=hGuQ8kuc9pgc9s8qqaq=dirpe0xb9q8qiLsFr0=vr0=vr0dc8meaabaqaciaacaGaaeqabaqabeGadaaakeaadaaeWaqaaiabdAeagnaaBaaaleaacqWGHbqyaeqaaaqaaiabdggaHjabg2da9iabigdaXaqaaiabikdaYiabicdaWaqdcqGHris5aOGaemOzay2aaSbaaSqaaiabdggaHbqabaaaaa@3926@ was added to Eq. 5 where *F*_*a *_is a regression parameter, and *f*_*a *_is the fraction of the amino acid type *a*. From a preliminary work based on a linear predictor [10], it was observed that this input slightly improved the accuracy by ~0.2%.

### Training and test data set

We carried out a 15-fold cross-validation test following exactly the same procedure and the same data set as the previous study [10]. In the data set, there are 680 protein domains, each of which represents a superfamily according to the SCOP database (version 1.65) [17]. This data set was randomly divided so that 630 domains were used for training and the remaining 50 domains for testing, and the random division was repeated 15 times [See Additional File 1]. No pair of these domains belong to the same superfamily, and hence they are not expected to be homologous. Thus, the present benchmark is a very stringent one. For obtaining PSSMs by running PSI-BLAST, we use the UniRef100 (version 6.8) amino acid sequence database [18] containing some 3 million entries. Also the number of iterations in PSI-BLAST homology searches was reduced to 3 times from 10 used in the previous study. This especially increased the accuracy of SS predictions. These results are consistent with the study of [19].

### Numerics

One drawback of the CRN method is the computational time required for numerically solving the equation of state (Eq. 4). For that purpose, instead of the Gauss-Seidel-like method previously used, we implemented a successive over-relaxation method which was found to be much more efficient. Let *v *denote the stage of iteration. We set the initial value of the state vectors (with *v *= 0) as

xi(0)=tanh⁡[αVui].     (7)
 MathType@MTEF@5@5@+=feaafiart1ev1aaatCvAUfKttLearuWrP9MDH5MBPbIqV92AaeXatLxBI9gBaebbnrfifHhDYfgasaacH8akY=wiFfYdH8Gipec8Eeeu0xXdbba9frFj0=OqFfea0dXdd9vqai=hGuQ8kuc9pgc9s8qqaq=dirpe0xb9q8qiLsFr0=vr0=vr0dc8meaabaqaciaacaGaaeqabaqabeGadaaakeaaieqacqWF4baEdaqhaaWcbaGaemyAaKgabaGaeiikaGIaeGimaaJaeiykaKcaaOGaeyypa0JagiiDaqNaeiyyaeMaeiOBa4MaeiiAaG2aamWaceaaiiGacqGFXoqycqWGwbGvcqWF1bqDdaWgaaWcbaGaemyAaKgabeaaaOGaay5waiaaw2faaiabc6caUiaaxMaacaWLjaWaaeWaceaacqaI3aWnaiaawIcacaGLPaaaaaa@4558@

Then, for *i *= 1, ..., *L *(in increasing order of *i*), we update the state vectors by

xi(2v+1)←xi(2v)+ω{tanh⁡[W(xi−1(2v+1)+xi+1(2v)+αVui]−xi(2v)}.     (8)
 MathType@MTEF@5@5@+=feaafiart1ev1aaatCvAUfKttLearuWrP9MDH5MBPbIqV92AaeXatLxBI9gBaebbnrfifHhDYfgasaacH8akY=wiFfYdH8Gipec8Eeeu0xXdbba9frFj0=OqFfea0dXdd9vqai=hGuQ8kuc9pgc9s8qqaq=dirpe0xb9q8qiLsFr0=vr0=vr0dc8meaabaqaciaacaGaaeqabaqabeGadaaakeaafaqaceGabaaabaacbeGae8hEaG3aa0baaSqaaiabdMgaPbqaaiabcIcaOiabikdaYiabdAha2jabgUcaRiabigdaXiabcMcaPaaakiabgcziSkab=Hha4naaDaaaleaacqWGPbqAaeaacqGGOaakcqaIYaGmcqWG2bGDcqGGPaqkaaGccqGHRaWkiiGacqGFjpWDcqGG7bWEcyGG0baDcqGGHbqycqGGUbGBcqGGObaAcqGGBbWwcqWGxbWvcqGGOaakcqWF4baEdaqhaaWcbaGaemyAaKMaeyOeI0IaeGymaedabaGaeiikaGIaeGOmaiJaemODayNaey4kaSIaeGymaeJaeiykaKcaaaGcbaGaey4kaSIae8hEaG3aa0baaSqaaiabdMgaPjabgUcaRiabigdaXaqaaiabcIcaOiabikdaYiabdAha2jabcMcaPaaakiabgUcaRiab+f7aHjabdAfawjab=vha1naaBaaaleaacqWGPbqAaeqaaOGaeiyxa0LaeyOeI0Iae8hEaG3aa0baaSqaaiabdMgaPbqaaiabcIcaOiabikdaYiabdAha2jabcMcaPaaakiabc2ha9jabc6caUaaacaWLjaGaaCzcamaabmGabaGaeGioaGdacaGLOaGaayzkaaaaaa@768C@

Next, we update them in the reverse order. That is, for *i *= *L*, ..., 1 (in decreasing order of *i*),

xi(2v+2)←xi(2v+1)+ω{tanh⁡[W(xi−1(2v+1)+xi+1(2v+2)+αVui]−xi(2v+1)}.     (9)
 MathType@MTEF@5@5@+=feaafiart1ev1aaatCvAUfKttLearuWrP9MDH5MBPbIqV92AaeXatLxBI9gBaebbnrfifHhDYfgasaacH8akY=wiFfYdH8Gipec8Eeeu0xXdbba9frFj0=OqFfea0dXdd9vqai=hGuQ8kuc9pgc9s8qqaq=dirpe0xb9q8qiLsFr0=vr0=vr0dc8meaabaqaciaacaGaaeqabaqabeGadaaakeaafaqaceGabaaabaacbeGae8hEaG3aa0baaSqaaiabdMgaPbqaaiabcIcaOiabikdaYiabdAha2jabgUcaRiabikdaYiabcMcaPaaakiabgcziSkab=Hha4naaDaaaleaacqWGPbqAaeaacqGGOaakcqaIYaGmcqWG2bGDcqGHRaWkcqaIXaqmcqGGPaqkaaGccqGHRaWkiiGacqGFjpWDcqGG7bWEcyGG0baDcqGGHbqycqGGUbGBcqGGObaAcqGGBbWwcqWGxbWvcqGGOaakcqWF4baEdaqhaaWcbaGaemyAaKMaeyOeI0IaeGymaedabaGaeiikaGIaeGOmaiJaemODayNaey4kaSIaeGymaeJaeiykaKcaaaGcbaGaey4kaSIae8hEaG3aa0baaSqaaiabdMgaPjabgUcaRiabigdaXaqaaiabcIcaOiabikdaYiabdAha2jabgUcaRiabikdaYiabcMcaPaaakiabgUcaRiab+f7aHjabdAfawjab=vha1naaBaaaleaacqWGPbqAaeqaaOGaeiyxa0LaeyOeI0Iae8hEaG3aa0baaSqaaiabdMgaPbqaaiabcIcaOiabikdaYiabdAha2jabgUcaRiabigdaXiabcMcaPaaakiabc2ha9jabc6caUaaacaWLjaGaaCzcamaabmGabaGaeGyoaKdacaGLOaGaayzkaaaaaa@7C08@

We then set *v *← *v *+ 1, and iterate Eqs. (8) and (9) until {**x**_*i*_} converges. The acceleration parameter of *ω *= 1.4 was found effective. The convergence criterion is

∑i=1L||xi(2v+2)−xi(2v+1)||RN/NL<10−3     (10)
 MathType@MTEF@5@5@+=feaafiart1ev1aaatCvAUfKttLearuWrP9MDH5MBPbIqV92AaeXatLxBI9gBaebbnrfifHhDYfgasaacH8akY=wiFfYdH8Gipec8Eeeu0xXdbba9frFj0=OqFfea0dXdd9vqai=hGuQ8kuc9pgc9s8qqaq=dirpe0xb9q8qiLsFr0=vr0=vr0dc8meaabaqaciaacaGaaeqabaqabeGadaaakeaadaaeWbqaaiabcYha8jabcYha8Hqabiab=Hha4naaDaaaleaacqWGPbqAaeaacqGGOaakcqaIYaGmcqWG2bGDcqGHRaWkcqaIYaGmcqGGPaqkaaGccqGHsislcqWF4baEdaqhaaWcbaGaemyAaKgabaGaeiikaGIaeGOmaiJaemODayNaey4kaSIaeGymaeJaeiykaKcaaOGaeiiFaWNaeiiFaW3aaSbaaSqaaiab=jfasnaaCaaameqabaGaemOta4eaaaWcbeaakiabc+caVmaakaaabaGaemOta4ealeqaaOGaemitaWKaeyipaWJaeGymaeJaeGimaaZaaWbaaSqabeaacqGHsislcqaIZaWmaaaabaGaemyAaKMaeyypa0JaeGymaedabaGaemitaWeaniabggHiLdGccaWLjaGaaCzcamaabmGabaGaeGymaeJaeGimaadacaGLOaGaayzkaaaaaa@5BE7@

where ||·||_**R**_^*N *^denotes the Euclidean norm. This criterion is much less stringent than previous study (10^-7^), but this does not affect the prediction accuracy significantly. Convergence is typically achieved within 10 to 12 iterations for one protein.

It is noted that the algorithm and parameters presented in this subsection are determined only for efficiently solving the equation of state (Eq. 4). As such, the choice of the parameters such as *ω *or the threshold of convergence has little, if any, impact on the prediction accuracy.

## Results and discussion

There are two main ingredients for the improved one-dimensional protein structure prediction in the present study. First is the use of large-scale critical random networks of 5000 dimension and 20 ensemble predictors. Second is the use of a large sequence database (UniRef100) for PSI-BLAST searches. As demonstrated in Table 1, the CRN method achieves remarkably accurate predictions. In comparison with the previous study [10] based on 2000-dimensional CRNs (10 ensemble predictors), the *Q*_3 _and *SOV *measures in SS predictions improved from 77.8% and 77.3% to 80.5% and 80.0%, respectively. Similarly, the average correlation coefficient improved from 0.726 to 0.746 for CN predictions, and from 0.601 to 0.613 for RWCO predictions. The 2-state predictions for CN yield, on average, *Q*_2 _= 76.8% per chain and 76.7% per residue, and Matthews' correlation coefficient of 0.533.

**Table 1:** Summary of average prediction accuracies per chain (median in parentheses).

SS	*Q*_3_= 80.5% (81.6)	*SOV *= 80.0% (81.1)
CN	*Cor *= 0.746 (0.768)	*DevA *= 0.686 (0.670)
RWCO	*Cor *= 0.613 (0.646)	*DevA *= 0.877 (0.812)

SS, Secondary structure prediction: *Q*_3 _is the percentage of correct prediction.; *SOV *is the segment overlap measure [28].

CN, Contact number prediction: *Cor *is the Pearson's correlation coefficient between the predicted and native CNs; *DevA *is the RMS error normalized by the standard deviation of the native CN [8].

RWCO, Residue-wise contact order prediction: *Cor *and *DevA *are defined as for CN but calculated with predicted and native RWCOs.

**Figure 1:**
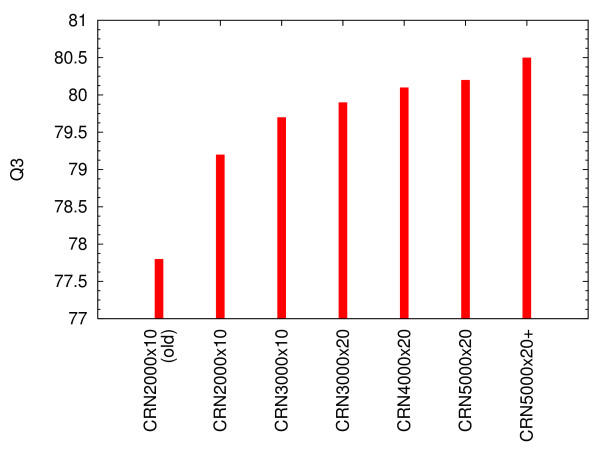
Dependence of SS prediction accuracy (*Q*_3_) on the dimension and ensemble size of CRNs. "CRN*n *× *m*" means an ensemble of *m *CRNs with *n*-dimensional state vectors. The suffix "+" indicates the use of the additional second stage filter. "CRN2000×10 (old)" is the result of the previous study [10].

The dependence of the SS prediction accuracy on the dimension and ensemble size of CRNs shows clearly that larger scale CRNs lead to better predictions (Fig. 1). The difference between "CRN2000×10 (old)" and "CRN2000×10" (i.e., both 10 sets of CRNs with 2000-dimensional state vectors) signifies the improvement due to the use of larger sequence database, which is in fact quite significant. On the other hand, the difference between CRN3000×10 and CRN3000×20 exemplifies the difference due to the the ensemble size (i.e., 10 vs. 20). Increasing the ensemble size does improve the accuracy, but the effect is relatively small. The accuracy steadily increases as we use 4000 and 5000 dimensional state vectors. Finally, a small improvement is made by the use of the second stage filter. It may be possible to employ even larger state vectors to further improve the accuracy, but we did not try such possibility because of the hardware limitations.

A closer examination of the SS prediction results (Table 2) reveals the drastic improvement of *β *strand prediction from *Q*_*E *_= 61.9% to 69.3% (per residue). Although the values of *Q*_*C *_and QEpre
 MathType@MTEF@5@5@+=feaafiart1ev1aaatCvAUfKttLearuWrP9MDH5MBPbIqV92AaeXatLxBI9gBaebbnrfifHhDYfgasaacH8akY=wiFfYdH8Gipec8Eeeu0xXdbba9frFj0=OqFfea0dXdd9vqai=hGuQ8kuc9pgc9s8qqaq=dirpe0xb9q8qiLsFr0=vr0=vr0dc8meaabaqaciaacaGaaeqabaqabeGadaaakeaacqWGrbqudaqhaaWcbaGaemyraueabaGaemiCaaNaemOCaiNaemyzaugaaaaa@3340@ are slightly lower than in the previous study by 0.6–1.0%, the accuracies of other classes have improved by 2.5–4%. Comparison of one prediction method with others is a difficult problem. Different methods are based on different data sets for both training and testing as well as the definition of secondary structural categories. In addition, prediction accuracies usually depend on the number of homologs used (see below), and the number of homologs, in turn, depends on the sequence database used. There are also ambiguities in what to compare: the best possible accuracies by any means or learning capacity of the method from a specified set of data, etc. With these cautions in mind, we present below the comparison between CRNPRED and other methods. The widely used PSIPRED program [4,20] which is based on conventional feed-forward neural networks achieves *Q*_3 _of 78%. A more recently developed method, Porter, [5] which is based on bidirectional recurrent neural networks achieves *Q*_3 _of 79%. An even more intricate method based on bidirectional segmented-memory recurrent neural networks [14] shows an accuracy of *Q*_3 _= 73% (this rather low accuracy may be attributed to the small size of the training set used). To further examine the performance of CRNPRED in comparison with other methods, we extracted recently solved protein structures (71 protein chains) from the EVA server [21] that do not include any of the proteins in the training set. For this purpose, we trained the parameters by using 2254 SCOP (version 1.65) domain representatives. The application of CRNPRED to these proteins yielded the average *Q*_3 _of 77.3%, whereas the values for the same set of proteins obtained by PSIPRED[20] and Porter[5] were 78.5% and 79.8%, respectively. That is, the CRNPRED result was inferior to these methods by 1–3% on the basis of the EVA data set. When the data set was limited to those 16 proteins with more than 300 homologs in the sequence database, the Q_3 _obtained by CRNPRED, PSIPRED, and Porter were 79.9%, 79.6%, and 80.9%, respectively. CRNPRED seems to be sensitive to the number of homologs used for constructing a PSSM. Regarding the contact number prediction, CRNPRED, achieving *Cor *= 0.75, is the most accurate method available today. The simple linear method [8] with multiple sequence alignment derived from the HSSP database [22] showed a correlation coefficient of 0.63. A more advanced method based on support vector machines (local window-based) achieves a correlation of 0.68 per chain[9].

**Table 2:** Summary of per-residue accuracies for SS predictions.

measure	*H*	*E*	*C*
*Q*_*s*_	82.7	69.3	84.0
Qspre MathType@MTEF@5@5@+=feaafiart1ev1aaatCvAUfKttLearuWrP9MDH5MBPbIqV92AaeXatLxBI9gBaebbnrfifHhDYfgasaacH8akY=wiFfYdH8Gipec8Eeeu0xXdbba9frFj0=OqFfea0dXdd9vqai=hGuQ8kuc9pgc9s8qqaq=dirpe0xb9q8qiLsFr0=vr0=vr0dc8meaabaqaciaacaGaaeqabaqabeGadaaakeaacqWGrbqudaqhaaWcbaGaem4CamhabaGaemiCaaNaemOCaiNaemyzaugaaaaa@339C@	84.4	78.9	78.3
*MC*	0.754	0.674	0.645

*Q*_*s*_: The number of correctly predicted residues of the SS class *s *= *H*, *E*, *C *divided by the number of residues in the class in native structures.

Qspre
 MathType@MTEF@5@5@+=feaafiart1ev1aaatCvAUfKttLearuWrP9MDH5MBPbIqV92AaeXatLxBI9gBaebbnrfifHhDYfgasaacH8akY=wiFfYdH8Gipec8Eeeu0xXdbba9frFj0=OqFfea0dXdd9vqai=hGuQ8kuc9pgc9s8qqaq=dirpe0xb9q8qiLsFr0=vr0=vr0dc8meaabaqaciaacaGaaeqabaqabeGadaaakeaacqWGrbqudaqhaaWcbaGaem4CamhabaGaemiCaaNaemOCaiNaemyzaugaaaaa@339C@: The number of correctly predicted residues of the SS class *s *= *H*, *E*, *C *divided by the number of residues predicted as the corresponding class.

*MC*: Matthews' correlation coefficient.

**Figure 2:**
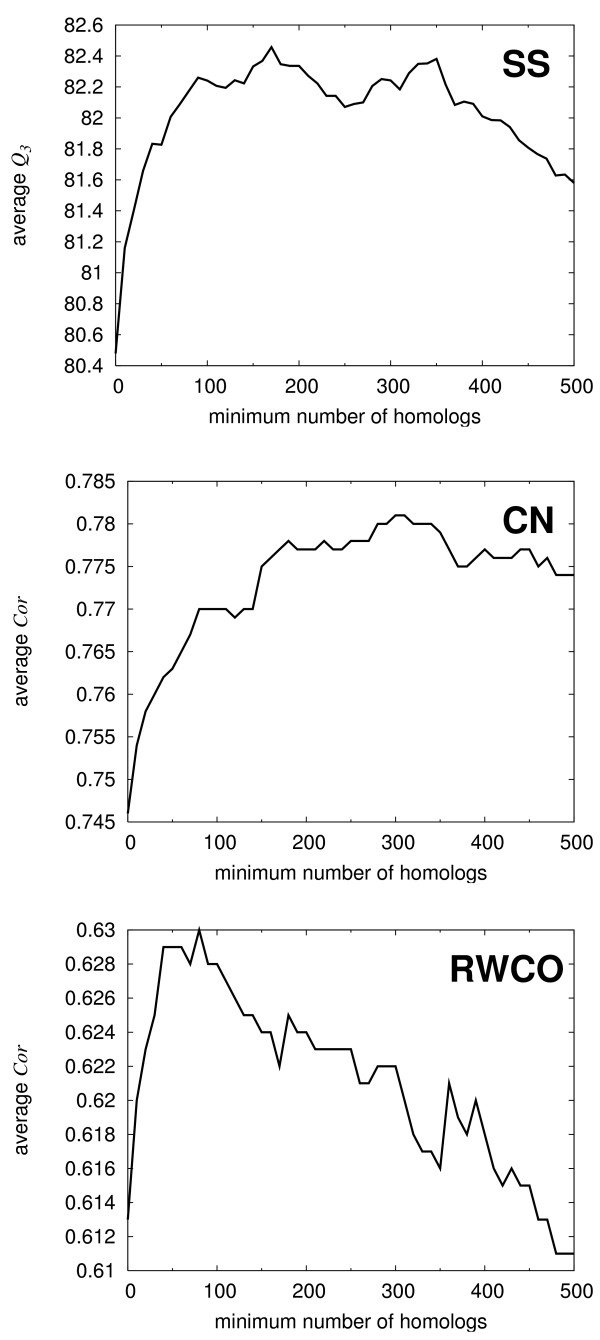
Average accuracy measure for given minimum number of homologs found by PSI-BLAST. From top to bottom: *Q*_3 _of secondary structure predictions, *Cor *of contact number predictions, and *Cor *of residue-wise contact number predictions.

It is known that the number of homologs found by the PSI-BLAST searches significantly affects the prediction accuracies [19]. We have examined this effect by plotting the accuracy measures for a given minimum number of homologs found by PSI-BLAST (Fig. 2). For example, we see in Fig. 2 that, for those proteins with more than 100 homologs, the average *Q*_3 _for SS predictions is 82.2%. The effect of the number of homologs significantly depends on the type of 1D structure. For SS prediction, *Q*_3 _steadily increases as the number of homologs increases up to 100, but it stays in the range between 82.0 and 82.4 until the minimum number of homologs reaches around 400, and then it starts to decrease. For CN prediction, *Cor *also increases steadily but more slowly, and it does not degrade when the minimum number of homologs reaches 500. This tendency implies that CN is more conservative than SS during protein evolution, which is consistent with previous observations [23,24]. On the contrary, RWCO exhibits a peculiar behavior. The value of *Cor *reaches its peak at the minimum number of homologs of 80 beyond which the value rapidly decreases. This indicates that RWCO is not evolutionarily well conserved. It was observed that the accuracies of SS and CN predictions constantly increased when the dimension of CRNs was increased from 2000 to 5000, but such was not the case for RWCO (data not shown). RWCO seems to be such delicate a quantity that it is very difficult to extract relevant information from the amino acid sequence.

Finally, we note on practical applicability of predicted 1D structures. We do not believe, at present, that the construction of a 3D structure purely from the predicted 1D structures is practical, if possible at all, because of the limited accuracy of the RWCO prediction. However, SS and CN predictions are very accurate for many proteins so that they may already serve as valuable restraints for 3D structure predictions. Also, SS and CN predictions may be applied to domain identification often necessary for experimental determination of protein structures. CRNPRED has been proved useful for such a purpose [25]. Although of the limited accuracy, predicted RWCOs still exhibit significant correlations with the correct values. Since RWCOs reflect the extent to which a residue is involved in long-range contacts, predicted RWCOs may be useful for enumerating potentially structurally important residues[26]. An interesting alternative application of the CRN framework is to regard the solution of the equation of state (Eq. 4) as an extended sequence profile. By so doing, it is straightforward to apply the solution to the profile-profile comparison for fold recognition [27]. Such an application may be also pursued in the future.

## Conclusion

We have developed the CRNPRED program that predicts secondary structures (SS), contact numbers (CN), and residue-wise contact orders (RWCO) of a protein given its amino acid sequence. The method is based on large-scale critical random networks. The achieved accuracies are at least as high as other predictors for SS and currently the best for CN and RWCO, although the success for RWCO prediction is still limited. CRNPRED will be a useful tool for computational as well as experimental biologists who need accurate one-dimensional protein structure predictions.

## Availability and requirements

**Project name: **CRNPRED

**Project home page: **

**Operating system: **UNIX-like OS (including Linux and Mac OS X).

**Programming language: **C.

**Other requirements: **zsh, PSI-BLAST (blastpgp), The UniRef100 amino acid sequence database.

**License: **Public domain.

**Any restrictions to use by non-academics: **None.

## Abbreviations

CRN, critical random network; SS, secondary structure; CN, contact number; RWCO, residue-wise contact order; 1D, one-dimensional; 3D, three-dimensional.

## Authors' contributions

A. R. K. designed and implemented the method, carried out benchmarks, wrote the first draft of the manuscript. A. R. K. and K. N. analyzed the results and improved the manuscript.

## Supplementary Material

Additional File 1 List of the SCOP domains used for cross-validation. (compressed (gzip) archive of text files). Click here for file
